# Evidence-based oral antiplatelet therapy among hospitalized Chinese patients with acute myocardial infarction: results from the Chinese acute myocardial infarction registry

**DOI:** 10.1186/s12872-021-02115-1

**Published:** 2021-06-14

**Authors:** Xiaofang Tang, Lifu Liu, Jingang Yang, Zhan Gao, Xueyan Zhao, Shubin Qiao, Runlin Gao, Zhifang Wang, Jinqing Yuan, Yuejin Yang

**Affiliations:** 1 Department of Cardiology, Fuwai Hospital, National Center for Cardiovascular Diseases, Chinese Academy of Medical Science and Peking Union Medical College, No. 167 North Lishi Road, Xicheng District, Beijing, 100037 China; 2 Department of Cardiology, The Central Hospital of Xinxiang, Xinxiang, Henan Province China

**Keywords:** Oral antiplatelet therapy, Acute myocardial infarction, Aspirin, P2Y12 inhibitors

## Abstract

**Background:**

Oral antiplatelet therapy is the cornerstone of treatment for acute myocardial infaction (AMI). However, detailed usage data on oral antiplatelet therapy are lacking.

**Methods:**

Using data from a nationally representative sample of patients with AMI, the detailed usage of oral antiplatelet therapy was analyzed in 40,202 consecutive eligible patients.

**Results:**

The proportions of patients with AMI taking loading doses of aspirin and P2Y12 inhibitors were relatively low (62.2% and 63.6%, respectively), whereas approximately 90% of patients received maintenance doses of aspirin, P2Y12 inhibitors, and dual antiplatelet therapy. The proportions of patients taking loading doses of aspirin and P2Y12 inhibitors gradually decreased with age. Male sex, an educational level of at least college, an interval from onset to treatment of < 24 h, and primary PCI use were associated with a higher proportion of patients taking a loading dose of antiplatelet therapy, whereas those receiving conservative treatment had a lower rate of antiplatelet use (all *P* < 0.05). The proportion of patients taking loading doses of aspirin was highest in the western region, and that of patients taking loading doses of P2Y12 inhibitors was highest in the eastern region (*P* < 0.05). In addition, 76.7% of patients with ST-elevation MI and 91% of patients with non-ST-elevation MI received 300-mg loading dose of clopidogrel.

**Conclusions:**

The proportion of patients with AMI receiving loading doses of aspirin and P2Y12 inhibitors during hospitalization was relatively low, and this rate was affected by many factors, such as age, sex, educational level, region of residence, and the interval from onset to treatment. The underutilization of guideline-based P2Y12 inhibitors was also problematic. Hence, quality improvement initiatives are needed to enhance adherence to guidelines to improve consistent use of oral antiplatelet therapy.

*Trial registration* The Chinese Acute Myocardial Infarction Registry; Trial registration number: ChiCTR-ONC-12002636; Registered 31 October 2012; http://www.chictr.org.cn/showproj.aspx?proj=6916

**Supplementary Information:**

The online version contains supplementary material available at 10.1186/s12872-021-02115-1.

## Background

Myocardial infarction (MI) remains a serious public health concern, and it has emerged as the leading cause of morbidity and mortality from cardiovascular disease around the world and in China, with hospitalization rates increasing significantly [1, 2]. Despite strong evidence of the benefits of both acute and longer-term management with antiplatelet therapy in patients with acute myocardial infarction (AMI), reports from Western countries indicated that physician compliance with guideline recommendations and the sustained use of antiplatelet therapy remain suboptimal and a challenging [3−7]. However, corresponding data (Evidence-based oral antiplatelet therapy among AMI patients during hospitalization and physician compliance with guideline recommendations of oral antiplatelet therapy**)** from China are scarce.

To date, few studies have explored this issue in China. The rate of aspirin use among patients with acute coronary syndrome (ACS) at the time of hospital discharge exceeded 90% between 2004 and 2006 in China according to the Clinical Pathways for Acute Coronary Syndromes in China (CPACS) study [8]. The early use of aspirin in patients with AMI increased over time in China (78.4% in 2001, 86.5% in 2006, and 90.0% in 2011), and the early rate of clopidogrel therapy for AMI in China increased from 45.7% in 2006 to 79.8% in 2011 according to the China PEACE-Retrospective AMI Study [9, 10].

However, detailed data regarding oral antiplatelet therapy such as the use of the loading and maintenance doses and receipt of dual antiplatelet therapy (DAPT) among Chinese patients with AMI in recent years are extremely limited. Thus, this study investigated the detailed use of oral antiplatelet therapy among hospitalized patients with AMI in a “real-world” contemporary era and determined whether oral antiplatelet use in patients with AMI accorded with current guidelines by analyzing a consecutive national sample derived from the Chinese acute myocardial infarction (CAMI) registry.

## Methods

### Study design

The CAMI registry is a prospective, nationwide, multicenter observational study of patients with AMI (NCT01874691). This project was approved by the institutional review board central committee at Fuwai Hospital, National Center for Cardiovascular Diseases of China (approval No.: 2012431) and conducted out in accordance with the tenets of the Declaration of Helsinki. Written informed consent was obtained from eligible patients before registration. Patient enrollment started in 2013. The registry covered all provinces and municipalities across mainland China (excluding Hong Kong and Macau), which made it likely representative of routine real-world clinical AMI care in China.

The treatment strategies for patients with ST-elevation myocardial infarction (STEMI) included primary percutaneous coronary intervention (PCI), thrombolysis, elective PCI, and conservative treatment. Primary PCI, defined as an emergent percutaneous catheter intervention in the setting of STEMI, without previous fibrinolytic treatment,was the preferred reperfusion strategy in patients with STEMI, provided that it can be performed expeditiously (i.e., within guideline-mandated times) by an experienced team and completed regardless of whether the patient presents to a PCI-capable hospital. Thrombolysis is an important reperfusion strategy, particularly in settings in which primary PCI cannot be offered to patients with STEMI within the recommended timeline. Elective PCI was defined as appropriate for percutaneous catheter intervention in the setting of STEMI and totally occluded arteries > 24 h after symptom onset in patients without signs of ischemia (regardless of whether fibrinolysis was performed). Conservative treatment was defined as treatment given to non-reperfused patients with STEMI that served as the only medications used as therapy [11, 12]. The treatment strategies for patients with non-STEMI (NSTEMI) included primary PCI, elective PCI, and conservative treatment. Primary PCI was defined as an early invasive strategy indicated in high-risk patients with NSTEMI who have refractory angina or hemodynamic or electrical instability (without serious comorbidities or contraindications to such procedures). Elective PCI was defined as a delayed invasive strategy for initially stabilized high-risk patients with UA/NSTEMI and those not at high risk. Conservative treatment was defined as treatment given to non-reperfused patients with NSTEMI that was the only medications used for treatment [13]. Clinical data, treatments, and outcomes were collected by local investigators and captured electronically using a fixed table, including a standardized set of variables and definitions, under rigorous data quality control. Complete details of the rationale and methodology of the CAMI registry study have been described elsewhere [14].

### Data collection

**Fig. 1:**
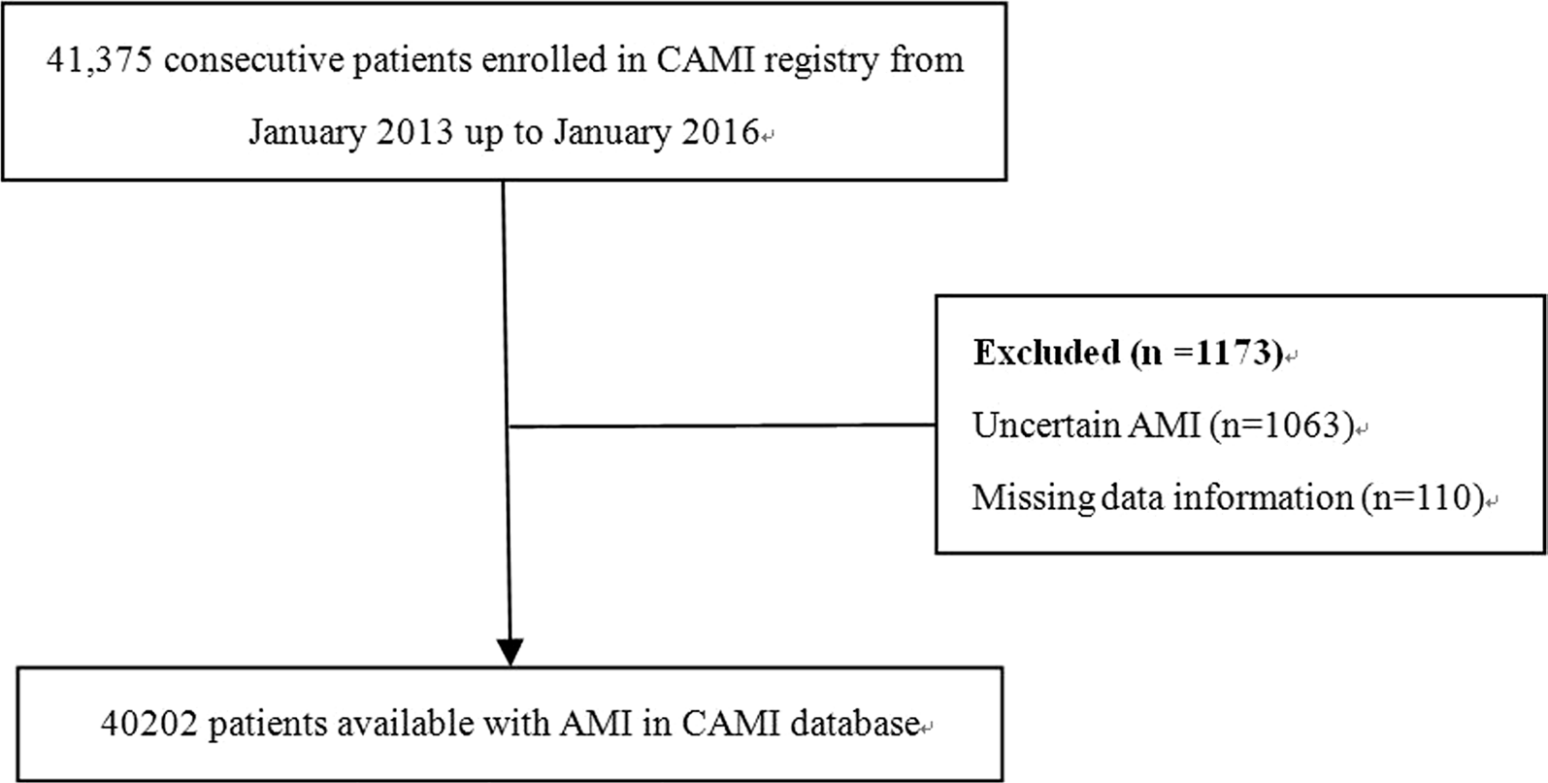
Flow chart for the study population

In the CAMI registry, eligible patients must be admitted within 7 days of acute ischemic symptoms with a primary clinical diagnosis of AMI, including STEMI or NSTEMI. The inclusion criteria must meet the guidelines of the third Universal Definition for Myocardial Infarction (2012). Types 1, 2, 3, 4b, and 4c are included, and types 4a and 5 are not eligible for the registry. From January 2013 to January 2016, 41,375 patients were enrolled. In the present study, 1063 patients with an uncertain AMI status and 110 patients with missing data were excluded sequentially. Finally, 40,202 eligible patients with AMI were included in the present study (Fig. 1).

### Statistical analyses

Patients’ baseline characteristics, medical history, treatments, and complications were evaluated. Continuous variables were presented as the mean ± standard deviation and compared using analysis of variance. Categorical variables were presented as counts and percentages and compared with the chi-squared test or Fisher’s exact test. Non-normally distributed variables were presented as the median and interquartile range and compared using Wilcoxon’s rank sum test. For all analyses, two-tailed *P* < 0.05 was considered statistically significant, and all analyses were performed using SAS version 9.4 (SAS Institute Inc., Cary, NC, USA).

## Results

A total of 40,202 patients diagnosed with AMI were consecutively enrolled in the CAMI registry from January 2013 to January 2016. Of these, 30,179 patients had STEMI, and 10,023 patients had NSTEMI. The baseline patient characteristics are presented in Table 1. Compared to the STEMI population, the NSTEMI population had higher a proportion of patients aged > 65 years; a higher rate of female sex; higher rates of previous MI, previous PCI, and previous coronary artery bypass surgery; and higher rates of comorbidities such as hypertension, hyperlipidemia, diabetes mellitus, peripheral arterial disease, heart failure, and stroke (all *P* < 0.001). The NSTEMI group also had lower rates of current smokers, onset-to-visit time < 24 h, and receipt of primary PCI (all *P* < 0.001).

**Table 1:** Baseline characteristics of the study participants

	Overall population (%/Mean ± SD)	STEMI (%/Mean ± SD)	NSTEMI (%/Mean ± SD)	P value
	N = 40,202	N = 30,179	N = 10,023	
*Demographics*				
Age (years)				< 0.001
< 55	11,786 (29.32%)	9475 (31.4%)	2311 (23.06%)	
55–65	11,116 (27.65%)	8562 (28.37%)	2554 (25.48%)	
65–75	10,005 (24.89%)	7218 (23.92%)	2787 (27.81%)	
> = 75	7295 (18.15%)	4924 (16.32%)	2371 (23.66%)	
Sex				< 0.001
Male	29,815 (74.16%)	22,963 (76.09%)	6852 (68.36%)	
Female	10,387 (25.84%)	7216 (23.91%)	3171 (31.64%)	
Access (n, %)				< 0.001
Emergency	4107 (10.52%)	3287 (11.24%)	820 (8.37%)	
External referral	22,944 (58.77%)	16,520 (56.49%)	6424 (65.56%)	
Family members or others Referral	9852 (25.24%)	8098 (27.69%)	1754 (17.9%)	
Hospital ward	444 (1.14%)	249 (0.85%)	195 (1.99%)	
Self-referral	1694 (4.34%)	1088 (3.72%)	606 (6.18%)	
Time from onset to treatment				< 0.001
< 24	28,664 (71.3%)	22,427 (74.31%)	6237 (62.23%)	
> 24 h	7873 (19.58%)	4918 (16.3%)	2955 (29.48%)	
Uncertain	3665 (9.12%)	2834 (9.39%)	831 (8.29%)	
Educational level (%)				0.0019
College or above	3097 (7.7%)	2396 (7.94%)	701 (6.99%)	
Below junior college	37,105 (92.3%)	27,783 (92.06%)	9322 (93.01%)	
Regions				< 0.001
Eastern	15,929 (39.62%)	11,577 (38.36%)	4352 (43.42%)	
Central	18,101 (45.03%)	13,961 (46.26%)	4140 (41.3%)	
western	6172 (15.35%)	4641 (15.38%)	1531 (15.27%)	
Medical history (n, %)				
Previous MI	2894 (7.2%)	1751 (5.8%)	1143 (11.4%)	< 0.001
Previous PCI	750 (1.87%)	496 (1.64%)	254 (2.53%)	< 0.001
Previous CABG	144 (0.36%)	60 (0.2%)	84 (0.84%)	< 0.001
Previous heart failure	938 (2.33%)	379 (1.26%)	559 (5.58%)	< 0.001
Previous stroke	3524 (8.77%)	2525 (8.37%)	999 (9.97%)	< 0.001
Previous peripheral arterial disease	271 (0.67%)	157 (0.52%)	114 (1.14%)	< 0.001
Bleeding history	459 (1.14%)	330 (1.09%)	129 (1.29%)	0.1188
Cardiovascular risk factors (n, %)				
Diabetes mellitus	7504 (18.67%)	5166 (17.12%)	2338 (23.33%)	< 0.001
Hypertension	19,661 (48.91%)	13,958 (46.25%)	5703 (56.9%)	< 0.001
Hyperlipidemia	2808 (6.98%)	1991 (6.6%)	817 (8.15%)	< 0.001
Family history of premature CAD	1237 (3.08%)	952 (3.15%)	285 (2.84%)	0.1151
Current smoker	18,661 (46.42%)	13,412 (44.44%)	5249 (52.37%)	< 0.001
Treatment strategy (n, %)				
Primary PCI	14,186 (35.29%)	12,998 (43.07%)	1188 (11.85%)	< 0.001
Elective PCI	9260 (23.03%)	6389 (21.17%)	2871 (28.64%)	< 0.001
Conservation	5149 (12.81%)	3580 (11.86%)	1569 (15.65%)	< 0.001

STEMI, ST-elevation myocardial infarction; NSTEMI, non-ST-elevation myocardial infarction; SD, standard deviation; MI, myocardial infarction; PCI, percutaneous coronary intervention; CABG, coronary artery bypass graft; CAD, coronary artery disease

The proportions of patients taking loading doses of aspirin and P2Y12 receptor inhibitors were both higher in the STEMI group than in the NSTEMI group (both *P* < 0.001). Conversely, the proportions of patients taking maintenance doses of aspirin and DAPT did not differ between the groups (Table 2).

**Table 2:** Detailed antiplatelet treatment in AMI patients

	Overall population (%/Mean ± SD)	STEMI (%/Mean ± SD)	NSTEMI (%/Mean ± SD)	P value
	N = 40,202	N = 30,179	N = 10,023	
Antiplatelet treatment strategy (n, %)				
Aspirin loading	25,011 (62.2%)	19,901 (65.9%)	5110 (51.0%)	< 0.001
P2Y12 loading	25,557 (63.6%)	19,993 (66.2%)	5564 (55.5%)	< 0.001
Aspirin maintenance	37,414 (93.1%)	28,087 (93.1%)	9327 (93.1%)	0.967
P2Y12 maintenance	36,405 (90.6%)	27,249 (90.3%)	9156 (91.3%)	0.002
DAPT	36,154 (89.9%)	27,134 (89.9%)	9020 (90.0%)	0.811

STEMI, ST-elevation myocardial infarction; NSTEMI, non-ST-elevation myocardial infarction; DAPT, dual antiplatelet treatment

### Detailed oral antiplatelet therapy

**Fig. 2:**
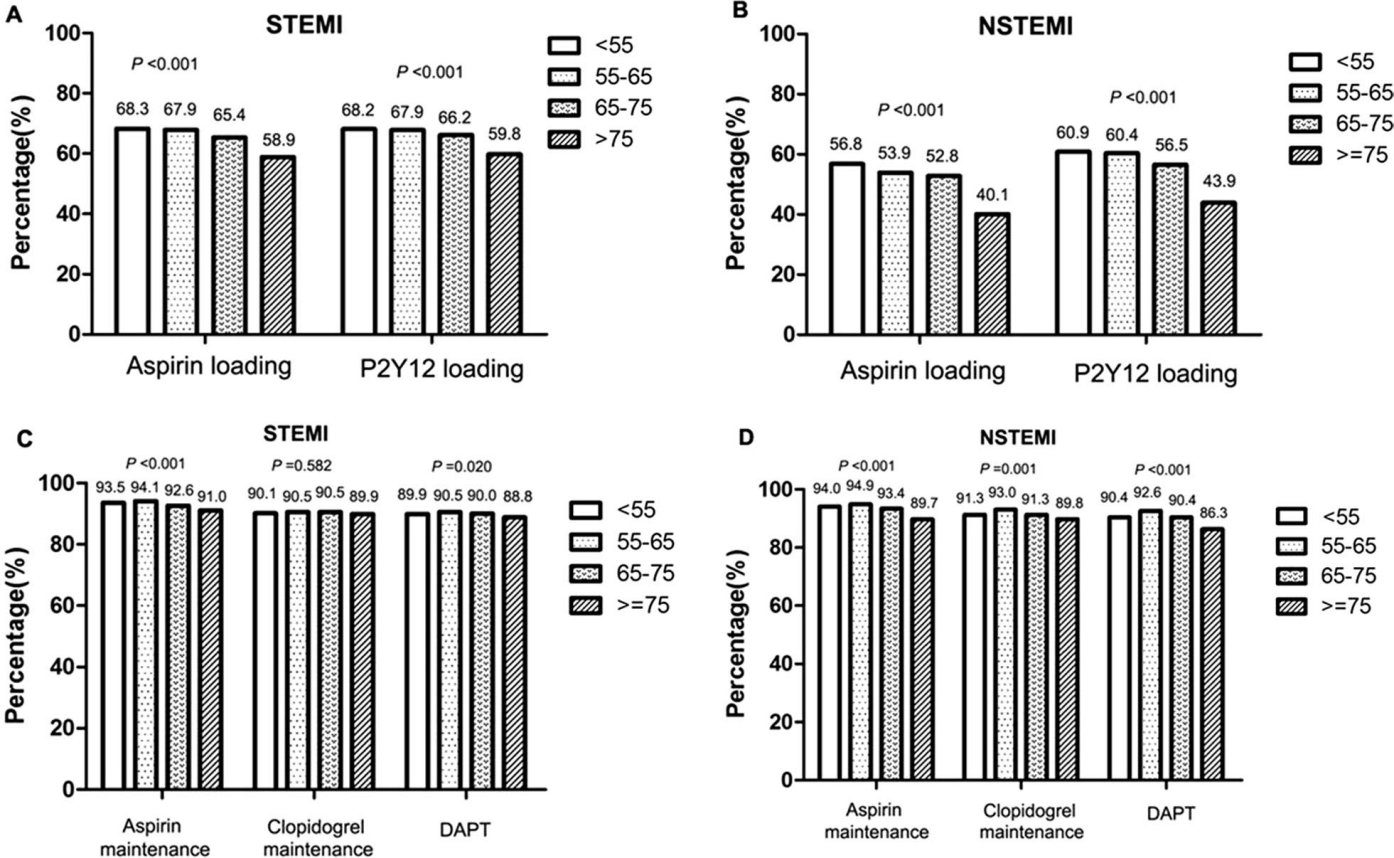
Comparisons of the rates of oral antiplatelet drug use by age in patients with acute myocardial infarction. **a** Loading doses of oral antiplatelet drugs by age in the STEMI group. **b** Loading doses of oral antiplatelet drugs by age in the NSTEMI group. **c** Maintenance doses of oral antiplatelet drugs by age in the STEMI group. **d** Maintenance doses of oral antiplatelet drugs by age in the NSTEMI group. STEMI, ST-elevation myocardial infarction; NSTEMI, non-ST-elevation myocardial infarction

The proportions of patients taking loading doses of aspirin and P2Y12 receptor inhibitors gradually decreased with age (both *P* < 0.05), especially among patients aged > 75 years (STEMI group: 58.9% and 59.8%, respectively; NSTEMI group: 40.1% and 43.9%, respectively). In the STEMI group, the proportions of patients taking maintenance doses of aspirin and DAPT were higher in patients age between 55 and 65 (94.1% and 90.5%, respectively) than in those > 75 years old (91.0% and 88.8%; respectively, both *P*); whereas the proportions of patients taking maintenance doses of P2Y12 receptor inhibitors did not differ between the age groups (94.1% vs. 91.0%; *P* = 0.582). Among the NSTEMI group, the proportions of patients taking maintenance doses of aspirin, P2Y12 receptor inhibitors, and DAPT were higher in patients aged 55–65 than in those aged > 75 years (94.9% vs. 89.7%, *P* < 0.001; 93.0% vs. 89.8%, *P* = 0.001; and 92.6% vs. 86.3%; *P* < 0.001; respectively, Fig. 2).

**Fig. 3:**
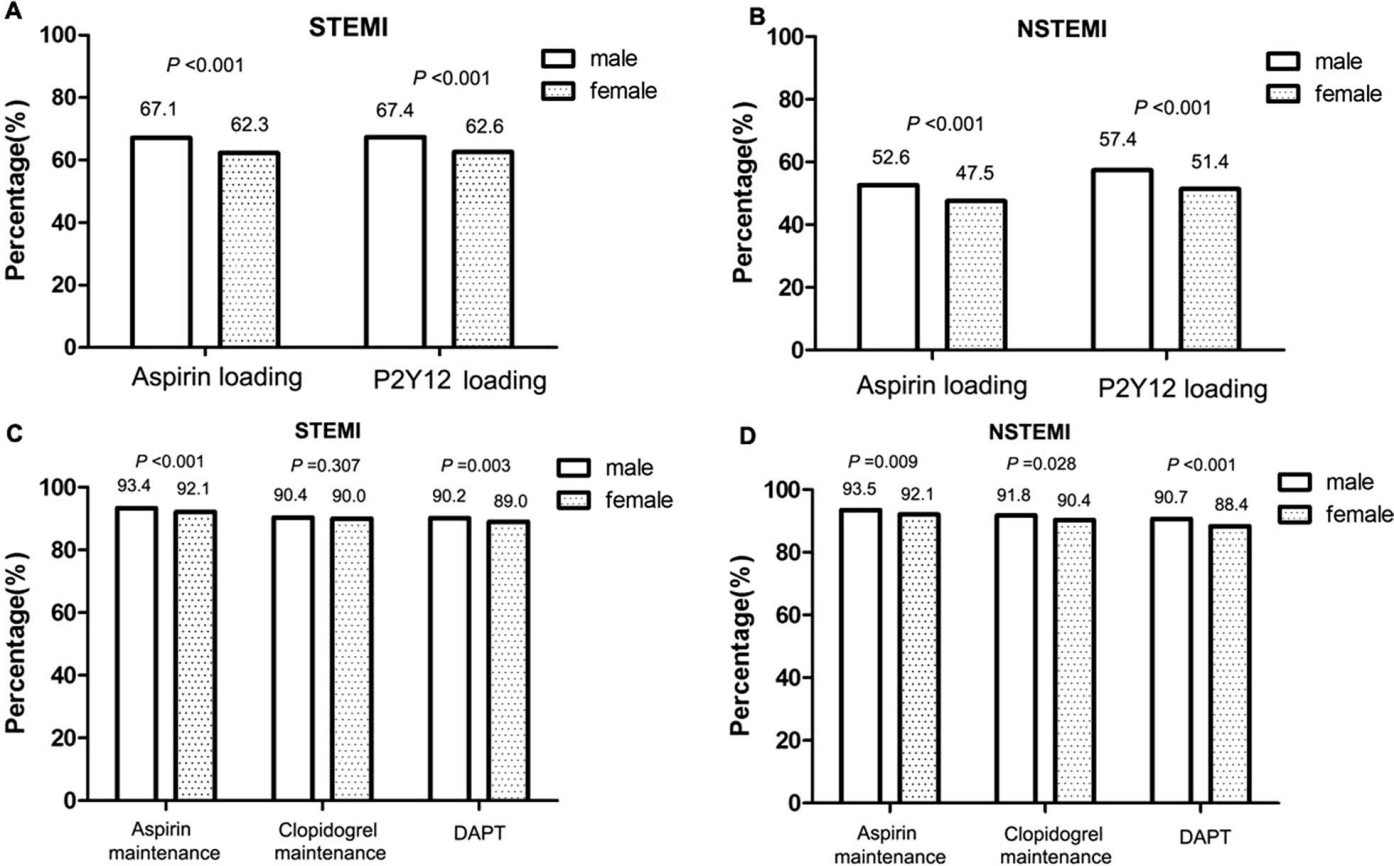
Comparisons of the rates of oral antiplatelet drug use by gender in patients with acute myocardial infarction. **a** Loading doses of oral antiplatelet drugs by gender in the STEMI group; **b** loading doses of oral antiplatelet drugs by gender in the NSTEMI group. **c** Maintenance doses of oral antiplatelet drugs by gender in the STEMI group. **d** Maintenance doses of oral antiplatelet drugs by gender in the NSTEMI group. STEMI, ST-elevation myocardial infarction; NSTEMI, non- ST-elevation myocardial infarction

The proportions of all enrolled patients of taking loading doses of aspirin and P2Y12 receptor inhibitors were both higher in men than in women (*P* < 0.05). In the STEMI group, the proportions of patients taking maintenance doses of aspirin and DAPT were both higher in men than in women (*P* < 0.05), but the rate of maintenance P2Y12 receptor inhibitor treatment did not differ between the sexes. In the NSTEMI group, the proportions of patients taking maintenance doses of aspirin, P2Y12 receptor inhibitors, and DAPT were all higher in men than in women (all* P* < 0.05, Fig. 3).

**Fig. 4:**
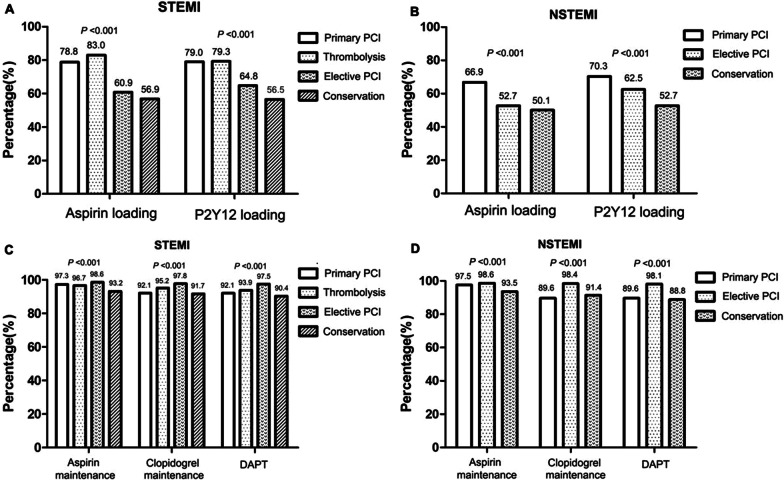
Comparisons of the rates of oral antiplatelet drug use in patients with acute myocardial infarction according to the treatment strategy. **a** Loading doses of oral antiplatelet drugs stratified by treatment strategy in the STEMI group. **b** Loading doses of oral antiplatelet drugs stratified by treatment strategy in the NSTEMI group. **c** Maintenance doses of oral antiplatelet drugs stratified by treatment strategy in the STEMI group. **d** Maintenance doses of oral antiplatelet drugs stratified by treatment strategy in the NSTEMI group. STEMI, ST-elevation myocardial infarction; NSTEMI, non-ST-elevation myocardial infarction

The proportions of patients taking loading and maintenance doses of aspirin, P2Y12 receptor inhibitors, and DAPT also differed according to the treatment strategies employed in patients with AMI (*P* < 0.05, Fig. 4). In STEMI group, the proportions of patients taking loading doses of aspirin and P2Y12 receptor inhibitors were relatively high, among those who received primary PCI (78.8% and 79.0%, respectively) and thrombolytic therapy (83% and 79.3%, respectively); conversely, the proportions were relatively low among patients who received elective PCI therapy (60.9% and 64.8%, respectively) and conservative therapy (56.9% and 56.5%, respectively). Meanwhile, the proportions of patients taking maintenance doses of aspirin and P2Y12 receptor inhibitors and DAPT were highest among those receiving elective PCI therapy (98.6%, 97.8% and 97.5%, respectively) and lowest among those receiving conservative therapy (93.2%, 91.7% and 90.4%, respectively). In the NSTEMI group, the proportions of patients taking loading doses of aspirin and P2Y12 receptor inhibitors were highest, among those receiving primary PCI (66.9% and 70.3%, respectively) and lowest among those receiving conservative therapy (50.1% and 52.7%, respectively). In addition, the proportions of patients taking maintenance doses of aspirin, P2Y12 receptor inhibitors, and DAPT were highest among those receiving elective PCI therapy (98.6%, 98.4% and 98.1%, respectively).

**Fig. 5:**
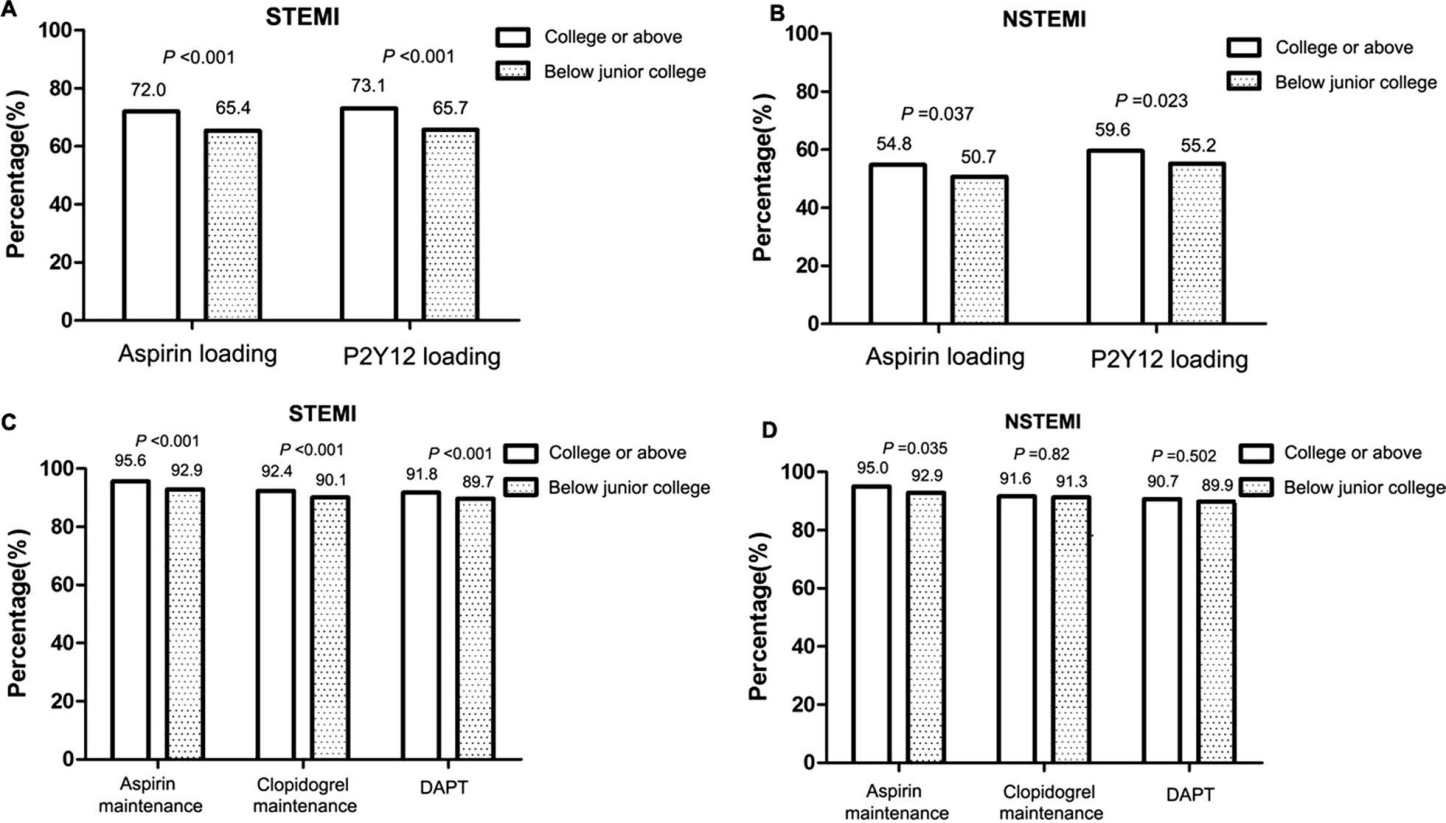
Comparisons of the rates of oral antiplatelet drug use by educational levels in patients with acute myocardial infarction. **a** Loading doses of oral antiplatelet drugs by educational level in the STEMI group. **b** Loading doses of oral antiplatelet drugs by educational level in the NSTEMI group. **c** Maintenance doses of oral antiplatelet drugs by educational level in the STEMI group. **d** Maintenance doses of oral antiplatelet drugs by educational level in the NSTEMI group. STEMI, ST-elevation myocardial infarction; NSTEMI, non-ST-elevation myocardial infarction

The proportions of patients taking loading doses of aspirin and P2Y12 receptor inhibitors were higher among those with at least a college education than in those with less education (*P* < 0.05, Fig. 5). In the STEMI group, the proportions of patients taking maintenance doses of aspirin, P2Y12 receptor inhibitors, and DAPT were all higher among those with at least a college education (all *P* < 0.05). Conversely, in the NSTEMI group, the proportion of patients taking maintenance doses of aspirin was higher among those with at least a college education (*P* < 0.05), whereas no difference in the use of maintenance doses of P2Y12 receptor inhibitors and DAPT were observed according to the educational level.

**Fig. 6:**
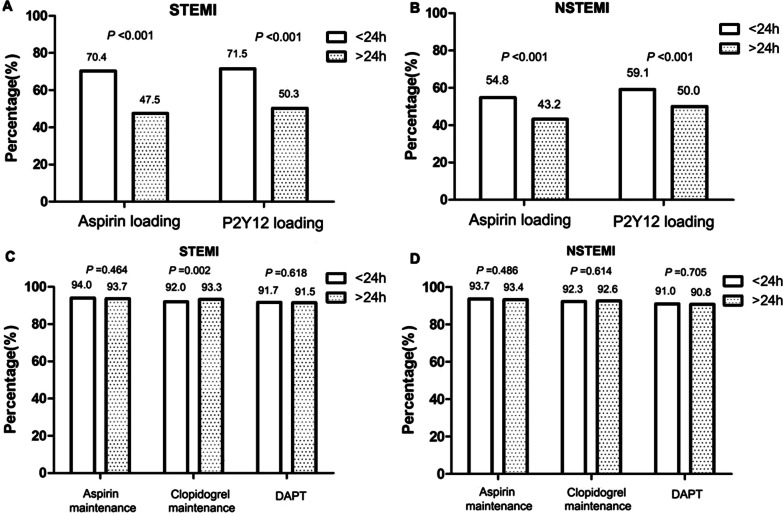
Comparisons of the rates of oral antiplatelet drug use by time from onset to treatment in patients with acute myocardial infarction. **a** Loading doses of oral antiplatelet drugs by the time from onset to treatment in the STEMI group. **b** Loading doses of oral antiplatelet drugs by the time from onset to treatment in the NSTEMI group. **c** Maintenance doses of oral antiplatelet drugs by the time from onset to treatment in the STEMI group. **d** Maintenance doses of oral antiplatelet drugs by the time from onset to treatment in the NSTEMI group. STEMI, ST-elevation myocardial infarction; NSTEMI, non-ST-elevation myocardial infarction

The proportions of patients taking loading doses of aspirin and P2Y12 receptor inhibitors were higher among those with an interval from onset to treatment of < 24 h than in those with a longer interval (*P* < 0.05). In both the STEMI and NSTEMI groups, the proportions of patients taking maintenance doses of aspirin and DAPT did not differ according to the interval from onset to treatment (Fig. 6).

**Fig. 7:**
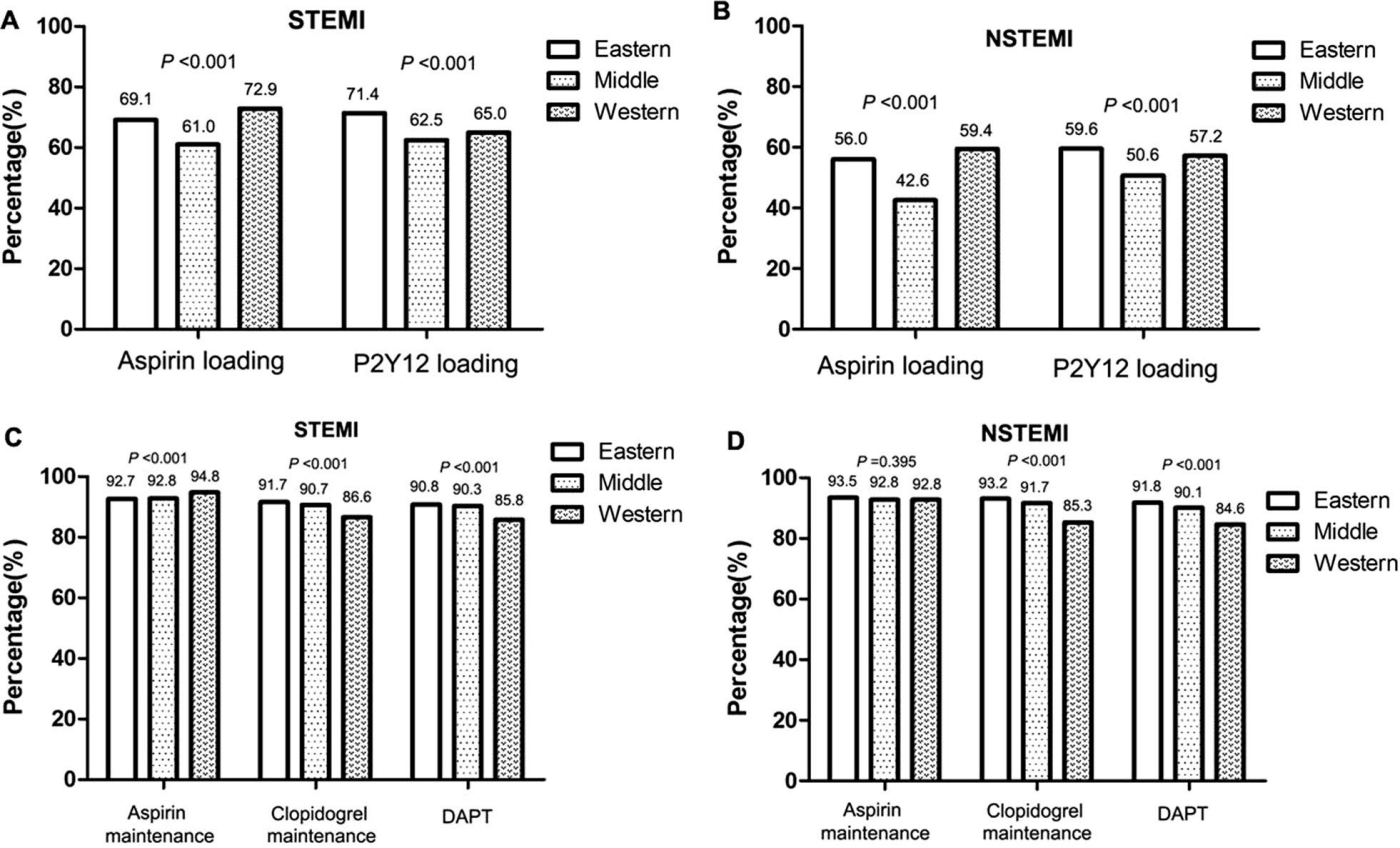
Comparisons of the rates of oral antiplatelet drug use by region of residence in patients with acute myocardial infarction. **a** Loading doses of oral antiplatelet drugs by region of residence in the STEMI group. **b** Loading doses of oral antiplatelet drugs by region of residence in the NSTEMI group. **c** Maintenance doses of oral antiplatelet drugs by region of residence in the STEMI group. **d** Maintenance doses of oral antiplatelet drugs by region of residence in the NSTEMI group. STEMI, ST-elevation myocardial infarction; NSTEMI, non-ST-elevation myocardial infarction

The proportions of patients taking loading and maintenance doses of aspirin, P2Y12 receptor inhibitors, and DAPT were differed according to the region of residence (*P* < 0.05, Fig. 7). Among the entire cohort, the proportion of patients taking loading doses of aspirin was highest in the western region, and lowest in the middle region**,** whereas the proportion of patients taking loading doses of P2Y12 receptor inhibitors was highest in the eastern region and lowest in the middle region (*P* < 0.05). Meanwhile, the proportion of patients taking maintenance doses of P2Y12 receptor inhibitors and DAPT was highest in the eastern region and lowest in the western region (*P* < 0.05).

### Utilization of P2Y12 receptor inhibitors

In the STEMI group, 76.7% of patients used a clopidogrel loading dose of 300 mg, 21.4% used a clopidogrel loading dose of 600 mg, and 1.9% used a ticagrelor loading dose of 180 mg. Moreover, 89.6% of patients used a clopidogrel maintenance dose of 75 mg/day, 8.3% used a clopidogrel maintenance dose of 100–150 mg/day, 0.38% used a clopidogrel maintenance dose of 25–50 mg/day, and 1.72% used a ticagrelor maintenance dose of 90–180 mg/day (Additional file 1: Fig. 1A and 1B).

In the NSTEMI cohort, 91% of patients used a clopidogrel loading dose of 300 mg, 8% used a clopidogrel loading dose of 600 mg, and 1% used a ticagrelor loading dose of 180 mg. Moreover, 92.7% of patients used a clopidogrel maintenance dose of 75 mg/day, 5.6% used a clopidogrel maintenance dose of 100–150 mg/day, 0.93% used a clopidogrel maintenance dose of 25–50 mg/day, and 0.77% used a ticagrelor maintenance dose of 90–180 mg/day (Additional file 2: Fig. 2A and 2B).

## Discussion

The major findings of this study were as follows: (1) The use of oral antiplatelet drugs during hospitalization had increased in Chinese patients with AMI in recent years. (2) In this study of a nationally representative sample of hospitalized patients in China, the usage of oral antiplatelet therapy by patients with AMI was affected by many factors, such as age, sex, educational level, regions, time since onset and treatment strategies. (3) Among patients with AMI, the most commonloading and maintenance doses of clopidogrel were 300 and 75 mg, respectively, from 2013 to 2016.

The epidemiological characteristics and real-world oral antiplatelet treatment of AMI have not been well studied in Asian countries despite its importance in public health. The CPACS registry found that 93% of all patients with ACS were prescribed aspirin, but only 44.6% of patients were prescribed clopidogrel at hospital discharge between September 2004 and May 2006 [8]. In the China-PACE study, the early use of aspirin in patients with AMI in China increased over time (78.4% in 2001, 86.5% in 2006, and 90.0% in 2011), and the early rate of clopidogrel therapy in Chinese patients with AMI significantly increased from 45.7% in 2006 to 79.8% in 2011 (*P* < 0.001) [9, 10]. In the present descriptive study, the proportions of patients with AMI who received maintenance doses of aspirin and clopidogrel both exceeded 90%, far exceeding the findings in prior studies [8–10, 15]. Meanwhile, almost 90% of patients with AMI were taking DAPT in our study, compared to rates in Taiwan of 65% in 2004 and 83.9% in 2008 [15].

In the present study, the utilization ratio of oral antiplatelet therapy in Chinese patients with AMI during hospitalization was affected by many factors, such as age, sex, educational level, region of residence, treatment strategy, and time from onset to treatment. As we know, longer time from onset to treatment may influence the selection of treatment strategys, and patients with lower educational levels may have worse economic conditions, which can also influence the choice of treatment. In our study, patients with lower educational level and a longer time from onset to treatment were less likely to receive oral antiplatelet therapy, especially loading doses of antiplatelet drugs. Meanwhile, patients who underwent primary PCI were more likely to receiving loading doses of oral antiplatelet therapy, patients who underwent elective PCI were more likely to receiving maintenance doses of oral antiplatelet therapy and DAPT, and patients who received conservative treatment were less likely to receiving oral antiplatelet therapy. Meanwhile, older and female patients were also less likely to receive oral antiplatelet therapy, especially loading doses of antiplatelet drugs, in our study. Economic development is imbalanced among the regions of China. In our study, the utilization rate of both loading and maintenance doses of P2Y12 receptor inhibitors was higher in the eastern region than that in other regions. The reason for this finding may be the relatively high price of P2Y12 receptor inhibitors based on the better economic condition in the eastern region.

In the clinic, individualized treatment with oral antiplatelet drugs is necessary to balance the risks of bleeding and ischemia within the context of the available evidence. Clinical factors increasing the risk of bleeding (e.g., age > 75 years old, female sex, prior stroke, body weight < 60 kg, renal dysfunction) and ischemia (e.g., diabetes mellitus, peripheral artery disease, prior cardiovascular event, prior coronary revascularization, left main stenting, bifurcation stenting) should be considered in the recommendation of oral antiplatelet therapy [7, 15–23]. In clinical practice, carefully selected patients may derive the greatest benefit from more intense antithrombotic therapy after AMI [24, 25].

In 2010, Chinese guidelines for patients with STEMI recommended an oral aspirin loading dose of 300 mg, followed by a maintenance dose of 100 mg/day, and an oral clopidogrel loading dose of 300 mg, followed by a maintenance dose of 75 mg/day [26]. The 2012 European Society of Cardiology (ESC) and 2013 American College of Cardiology (ACC)/American Heart Association (AHA) guidelines for STEMI recommended an oral clopidogrel loading dose of 600 mg orally, followed by a maintenance dose of 75 mg/day, whereas the recommendation for ticagrelor was an oral loading dose of 180 mg, followed by a maintenance dose of 90 mg b.i.d [11, 12]. The 2012 ACCF/AHA guideline for UA/NSTEMI also mentioned new P2Y12 inhibitors, including ticagrel [13]. It was not until 2015 that the ticagrelor was first recommended in the updated Chinese STEMI guidelines, which recommended an oral clopidogrel loading dose of 600 mg, followed by a maintenance dose of 75 mg/day and an oral ticagrelor loading dose of 180 mg, followed by a maintenance dose of 90 mg b.i.d [27].

The patients in our study were enrolled between 2013 and 2016. Among the patients with AMI, the most common loading and maintenance doses of clopidogrel were 300 and 75 mg, respectively. Because this study was descriptive in nature, we could not identify the reasons for the usage habits of P2Y12 receptor inhibitors. In the ESC 2012 or ACC 2013 guidelines, the loading dose of clopidogrel was initially recommended as 600 mg; however, the Chinese guidelines only updated the high loading dose of clopidogrel in 2015, which may partly explain the use of a low clopidogrel loading dose of 300 mg. At the same time, it was only in 2015 that the Chinese guidelines recommended the use of ticagrelor, which may explain the low use of this drug. In addition, the common reason for the low use of high-dose loading clopidogrel and ticagrelor is the risk of bleeding. The ACC/AHA guidelines for STEMI and NSTEMI are widely upheld in China, and China's own guidelines are widely recognized; however, these findings suggest a significant gap between evidence and practice, which may, in large part, be attributable to a lack of familiarity with current treatment recommendations. The PROGRESS trial investigated the adherence to ticagrelor therapy in patients after ACS [28]. Adherence to antithrombotic treatment in patients with AMI is extremely important, hence, increasing the prescription rate of guideline-based medications through quality improvement programs may be necessary to enhance adherence to AMI treatment guidelines. In addition to antithrombotic drug treatment, the control of other risk factors, particularly LDL and Lp(a), which were considered to be the independent risk factors of atherosclerotic cardiovascular disease, especially in patients with AMI, is also important [29–31].

## Limitations

As the first prospective national registry study of patients with AMI in China, the CAMI registry study revealed current problems in the Chinese health care system. Data were valuable, specific, and updated, and they were based on a large population base. The CAMI study has five steps to support data quality control in the registry [14]. Nevertheless, our study had several limitations. First, the CAMI study was subject to inherent limitations and potential biases, including the collection of non-randomized data, missing or incomplete information, and potential confounding by drug indications or other unmeasured covariates that must be considered in result interpretation. Second, we did not include follow-up data with oral antiplatelet drugs after hospital discharge. Future research plans to further explore how to follow guidelines to improve the usability and compliance of antiplatelet drug therapy.

## Conclusions

There was a progressive improvement in the use of oral antiplatelet treatment for patients with AMI in China, and this improvement was also affected by many factors, such as age, sex, educational levels, and treatment strategies. However, under utilization of the guideline-based P2Y12 receptor inhibitors for AMI is a major problem in China. The guideline-based oral antiplatelet therapies in patients with AMI in China present an important clinical implication; thus, national policies and initiatives are necessary to enhance adherence to the guidelines to improve oral antiplatelet therapy.

## Supplementary Information

**Additional file 1:** **Figure 1**. Rate of loading doses of P2Y12 receptor inhibitor use among patients with acute myocardial infarction. A, loading doses in the STEMI group. B, loading doses in the NSTEMI group. STEMI, ST-elevation myocardial infarction; NSTEMI, non- ST-elevation myocardial infarction

**Additional file 2:** **Figure 2**. Rate of maintenance doses of P2Y12 receptor inhibitor use among patients with acute myocardial infarction. A, maintenance doses in the STEMI group; B, maintenance doses in the NSTEMI group. STEMI, ST-elevation myocardial infarction; NSTEMI, non- ST-elevation myocardial infarction

## Data Availability

The dataset analyzed in the current study is not publicly available because of the lack of consent from the study participants, but it is available from the corresponding author on reasonable request for researchers who meet the criteria for access to confidential data.
