# Supplementary figures and images for: Evidence-based oral antiplatelet therapy among hospitalized Chinese patients with acute myocardial infarction: results from the Chinese acute myocardial infarction registry
Source: BMC Cardiovasc Disord. 2021 Jun 14;21:299. doi: 10.1186/s12872-021-02115-1 (PMC8204547; doi:10.1186/s12872-021-02115-1)

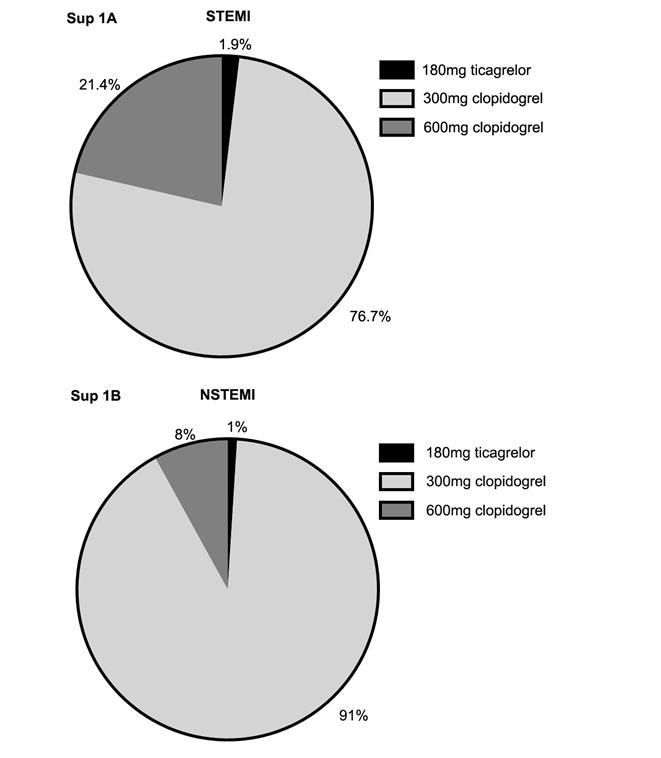

Supplement: Supplementary file 1 — Additional file 1: Figure 1. Rate of loading doses of P2Y12 receptor inhibitor use among patients with acute myocardial infarction. A, loading doses in the STEMI group. B, loading doses in the NSTEMI group. STEMI, ST-elevation myocardial infarction; NSTEMI, non- ST-elevation myocardial infarction [file 12872_2021_2115_MOESM1_ESM.tif]

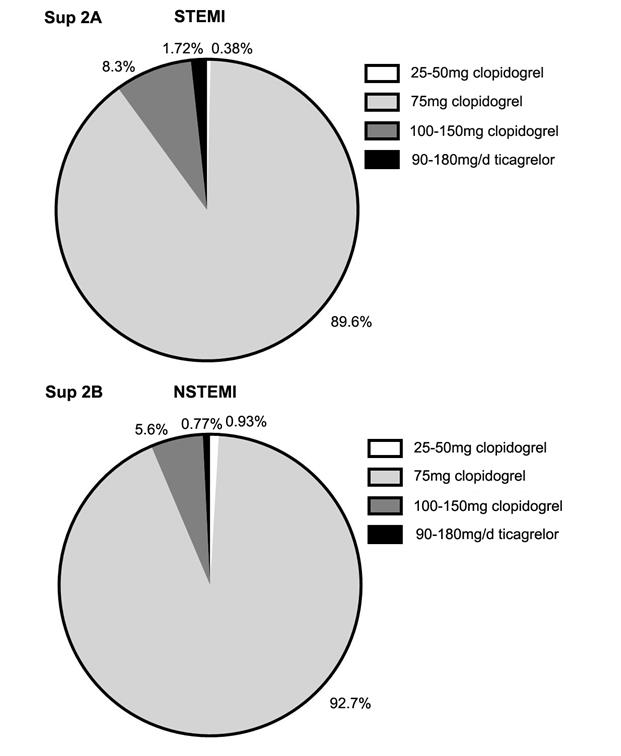

Supplement: Supplementary file 2 — Additional file 2: Figure 2. Rate of maintenance doses of P2Y12 receptor inhibitor use among patients with acute myocardial infarction. A, maintenance doses in the STEMI group; B, maintenance doses in the NSTEMI group. STEMI, ST-elevation myocardial infarction; NSTEMI, non- ST-elevation myocardial infarction [file 12872_2021_2115_MOESM2_ESM.tif]
